# Nicotine and tobacco smoke reshape neuronal signaling networks from a single-nucleus transcriptomic perspective

**DOI:** 10.3389/fnins.2026.1953804

**Published:** 2026-10-07

**Authors:** Jian Jiang, Anfu Hu, Yixiao Jin, Xia Li, Guo-Jun Zhou, Yi Shen, Chengyun Xu, Xing Ji, Ximei Wu, Hong-Juan Wang

**Affiliations:** 1 China Tobacco Zhejiang Industrial Co., Ltd., Hangzhou, China; 2 China Tobacco Sichuan Industrial Co., Ltd., Chengdu, China; 3 Department of Pharmacology, Hangzhou City University School of Medicine, Hangzhou, China; 4 Shulan International Medical College, Zhejiang Shuren University, Hangzhou, China; 5 Department of Pharmacology, Zhejiang University School of Medicine, Hangzhou, China; 6 China National Tobacco Quality Supervision and Test Center, Zhengzhou, China

**Keywords:** nicotine, NRXN pathway, smoke exposure, snRNA-seq, VTA

## Abstract

Nicotine and cigarette smoke exposure significantly impact brain function, yet their distinct cellular and molecular mechanisms remain unclear. To address this, we conducted comprehensive single-nucleus RNA sequencing (snRNA-seq) analysis on whole-brain samples and specifically on the ventral tegmental area (VTA) of adult C57BL/6 mice following nicotine or smoke exposure. Our study identified 18 major transcriptionally distinct cell populations across the whole brain and 27 distinct clusters within the VTA, covering major neuronal and glial cell types. Differential gene expression and KEGG pathway enrichment analyses revealed distinct transcriptional responses: nicotine exposure was associated with enrichment of synaptic and addiction-related transcriptional pathways in neuronal populations, including dopaminergic and glutamatergic pathways, whereas smoke exposure was associated with broader enrichment of metabolic-, immune-, and endocrine-related pathways. CellChat predicted condition-associated communication differences, including higher inferred NRXN communication from excitatory neurons to oligodendrocytes and other excitatory neurons under nicotine exposure. Smoke exposure also increased oligodendrocyte PISD expression, a transcriptomic change potentially associated with mitochondrial phospholipid metabolism. Collectively, these data define cell-type-specific transcriptional and predicted communication signatures of nicotine and smoke exposure that require functional validation.

## Introduction

Nicotine, the principal addictive component of tobacco, exerts profound effects on the central nervous system by modulating neuronal circuits involved in reward, motivation, and reinforcement (Kim and Picciotto, 2023; Benowitz, 2008; Picciotto and Kenny, 2021; Wittenberg et al., 2020; Wills et al., 2022). The ventral tegmental area (VTA), a key region in the mesolimbic dopamine system, plays a pivotal role in mediating these effects (Morales and Margolis, 2017; Wang et al., 2025; Liu et al., 2022). While previous studies have explored the impact of nicotine on the VTA, the precise cellular and molecular mechanisms underlying nicotine-induced neuroadaptations remain incompletely understood (Zell et al., 2020; Nguyen et al., 2021; Bariselli et al., 2016). Recent advancements in single-cell RNA sequencing (scRNA-seq) have enabled the dissection of complex brain regions at unprecedented resolution, allowing for the identification of distinct neuronal subtypes and their transcriptional profiles (Tiklová et al., 2019; Poulin et al., 2020). Applying scRNA-seq to the VTA has revealed diverse neuronal populations, including dopaminergic, GABAergic, and glutamatergic neurons, each contributing uniquely to the processing of rewarding stimuli (Zell et al., 2020; Phillips et al., 2022; Oriol et al., 2025). While several studies have explored nicotine-induced changes using purified compounds or *in vitro* models, the specific transcriptional alterations induced by whole cigarette smoke exposure remain poorly characterized at single-nucleus resolution. Unlike purified nicotine exposure, whole cigarette smoke represents a complex mixture containing numerous interacting constituents, making it difficult to attribute observed transcriptional responses to any single component.

Because synaptic organization and neuronal communication are closely involved in addiction-related neural adaptations, synaptic adhesion molecules represent an important molecular link for understanding exposure-associated changes in neuronal signaling. Among these molecules, neurexins (NRXNs) are presynaptic transmembrane proteins that interact with neuroligins and other partners to regulate synapse organization, specificity, and stability (Song et al., 1999; Liu et al., 2022). Among the NRXN family, NRXN1 and NRXN3 have been genetically associated with substance use disorders (Bierut et al., 2007; Nussbaum et al., 2008). Several human studies have identified NRXN3 polymorphisms associated with nicotine and alcohol dependence, as well as behavioral traits such as impulsivity, which are known risk factors for addiction (Novak et al., 2009). These findings suggest that NRXN3 may influence addiction vulnerability through its modulation of synaptic connectivity and neuronal communication.

In this study, we employed snRNA-seq to profile the transcriptomic landscape of the VTA and whole brain in mice subjected to nicotine exposure, cigarette smoke exposure, and control conditions. Our analysis focused on delineating the neuronal subtypes affected by these exposures and identifying molecular pathways associated with exposure-induced transcriptional responses, with particular attention to NRXN-related signaling and its cell-type-specific patterns. By integrating single-nucleus transcriptomic data with pathway analysis, we aim to characterize exposure-associated cellular and molecular signatures relevant to nicotine and cigarette smoke responses.

## Materials and methods

### Mice treatments

C57BL/6 mice were purchased from Shanghai SLAC Laboratory Animal Co. Ltd. (Shanghai, China) and were subjected to two treatment protocols. For pharmacological exposure, six mice received daily subcutaneous injections of nicotine (purity: 99.5%, Heno Bioengineering Co. Ltd., Enshi City, China) at 1 mg/kg for 14 consecutive days. For smoke exposure, six mice were exposed to mainstream smoke of cigarettes (#2 standard cigarettes, China National Tobacco Co., Zhengzhou, China) at a particulate concentration of 1,000 mg/m^3^ via whole-body exposure for 2 h daily and for 14 consecutive days. Six control mice received normal saline injection and fresh air exposure. Mice were sacrificed 1 h after the last injection or exposure, and 3 whole brains and 3 VTAs in each group were harvested and pooled for snRNA-seq analysis, respectively.

The mice used in this study were housed in a specific pathogen-free (SPF) room under a 12–12 h light–dark cycle with constant temperature and food and water provided ad libitum at the Hangzhou City University Animal Care Facility. The mice were euthanized by CO_2_ inhalation and subsequent cervical dislocation. All experimental procedures were approved by the Institutional Animal Care and Use Committee of Hangzhou City University (Approval No. 25090). All methods were performed in accordance with relevant guidelines and regulations and in accordance with ARRIVE guidelines.

### Determination of nicotine contents in serum

To quantify nicotine levels in serum, we employed liquid chromatography–tandem mass spectrometry (LC–MS/MS, Agilent1260-6420, Agilent Technologies, Inc., Santa Clara, CA) following tissue extraction. Briefly, mice were euthanized 1 h after the final exposure to nicotine or cigarette smoke, and blood samples were collected for serum preparation. After clotting, blood samples were centrifuged to obtain serum, which was stored at −80 °C until analysis. For nicotine extraction, serum samples were mixed with 80% methanol containing nicotine-d₄ (10 ng/mL) as an internal standard. The mixtures were vortexed, sonicated for 10 min, and centrifuged at 15,000 × g for 15 min at 4 °C. The supernatants were collected and filtered through a 0.22 μm membrane before LC–MS/MS analysis.

Nicotine was quantified using an Agilent 1,290 UHPLC system coupled to an Agilent 6,460 triple-quadrupole mass spectrometer operated in positive electrospray ionization (ESI) mode. Chromatographic separation was performed on a C18 reversed-phase column (2.1 × 100 mm, 1.8 μm particle size; Agilent) using water containing 0.1% formic acid and acetonitrile containing 0.1% formic acid as mobile phases under gradient elution. Nicotine and nicotine-d₄ were monitored in multiple reaction monitoring (MRM) mode using the transitions m/z 163 → 130 and m/z 167 → 134, respectively. Serum nicotine concentrations were determined from a calibration curve using the nicotine-to-nicotine-d₄ peak area ratio and expressed as ng/mL.

### Isolation of brain nuclei

For snRNA-seq, tissues from three individual mice within each experimental group were pooled prior to nuclei isolation. Three whole-brain samples were pooled to generate one whole-brain sample per condition, while three independently dissected VTA tissues were pooled to generate one VTA sample per condition. Each pooled tissue sample was subsequently processed as a single sample for nuclei isolation and library preparation. Individual animals were therefore not sequenced as independent biological replicates.

Brain nuclei isolation was performed as follows, brain tissues were finely minced into 0.1 cm fragments and transferred into a mixture of 1 mL lysis buffer and 1 mL PBS. Samples were washed twice in PBS by centrifugation to remove the supernatant. Tissues were then incubated with 2 mL of pre-chilled lysis buffer on ice for 2 min for pre-lysis. Homogenization was carried out using a Dounce homogenizer (Sigma, St. Louis, MO), followed by gentle pipetting (5–6 times) to ensure complete dissociation. The homogenate was kept on ice for 6 min, after which 2 mL of pre-chilled 4% BSA was added to dilute the mixture and terminate lysis. This was followed by pipetting (6–8 times) with a Pasteur pipette. Samples were centrifuged at 300 × g for 5 min at 4 °C, and the supernatant was discarded. The resulting pellet was resuspended in 2 mL of lysis buffer containing 4% BSA and incubated on ice for an additional 3 min. Cellular debris was removed using the Miltenyi Biotec Debris Removal Solution (Order no. 130–109-398). After a second centrifugation under the same conditions, the nuclei were resuspended in a nuclear resuspension buffer (1 × PBS, 2% BSA, 0.1% RNase inhibitor, Roche). Nuclei were then filtered through a 20-μm cell strainer and counted.

### Single-nucleus RNA-sequencing

For each tissue type and experimental condition, the pooled nuclei suspension described above was used to generate a single snRNA-seq library. Single-nucleus RNA-sequencing was performed using the Chromium Single Cell 3′ v3.1 Reagent Kit (10 × Genomics, Pleasanton, CA), following the manufacturer’s instructions. Approximately 8,000 nuclei were loaded per channel on a Chromium chip. Subsequent cDNA amplification and library preparation were carried out as per the standard protocol. Final libraries were sequenced using an Illumina NovaSeq 6,000 platform with paired-end 150 bp reads by LC-Bio Technology Co. (Hangzhou, China).

### Data processing and quality control

Raw sequencing data were processed using Cell Ranger (v6.1.2, 10 × Genomics) to generate gene expression matrices. Further quality control and normalization were performed in Seurat (v5.0.0) (Hao et al., 2021). Low-quality nuclei with fewer than 200 genes or >5% mitochondrial gene content were excluded. Expression matrices from the three conditions were merged and jointly analyzed. No explicit batch-correction algorithm, including Harmony (Korsunsky et al., 2019), was applied because all libraries were generated and sequenced within the same experimental batch.

### Dimensionality reduction and clustering

Principal component analysis (PCA) was performed on the top 2000 variable genes, followed by UMAP for visualization. Clustering was conducted using the Louvain algorithm with resolution set to 0.5–1.0, depending on the dataset. Clusters were annotated using canonical marker genes. A common broad cell-class marker framework was applied to both the whole-brain and VTA datasets, while clustering was performed independently for the two tissue datasets. Within each dataset, nuclei from control, nicotine, and smoke conditions were clustered jointly so that cluster identities were shared across conditions.

### Differential expression analysis

Differential expression analysis was performed separately within each annotated cell type or cluster using the Wilcoxon rank-sum test implemented in Seurat (v5.0.0). For each cell type or cluster, nuclei from the nicotine-treated or smoke-exposed condition were compared with nuclei from the corresponding control condition using the FindMarkers function. Differential-expression testing was performed using the normalized RNA expression data and not on dimensionality-reduction coordinates or batch-corrected expression values.

Differentially expressed genes (DEGs) were defined as genes with an absolute log2 fold change (|log2FC|) > 0.25 and a Benjamini-Hochberg-adjusted *p*-value < 0.05. Genes with log2FC > 0.25 were classified as upregulated, whereas genes with log2FC < −0.25 were classified as downregulated. Because tissues from three animals per condition were pooled before nuclei isolation and only one pooled library was generated per tissue and condition, independent animal-level biological replicates were unavailable; therefore, replicate-aware pseudobulk analysis could not be performed, and nucleus-level DEG results were interpreted as exploratory.

### KEGG pathway enrichment analysis

To evaluate biological pathway enrichment, KEGG (Kyoto Encyclopedia of Genes and Genomes) analysis was performed using the R package clusterProfiler (v4.4.4) (Kanehisa and Goto, 2000; Kanehisa et al., 2025). DEGs from each cell cluster or condition were submitted to enrichKEGG with organism set to “mmu” (*Mus musculus*). Pathways with adjusted *p*-value < 0.05 were considered significantly enriched. Results were visualized using barplots, dotplots, and enrichment maps. Gene set enrichment analysis (GSEA) was also conducted using the fgsea package with curated addiction and metabolism-related gene sets from the KEGG database.

### Cell–cell communication analysis

To infer potential intercellular communication patterns, we applied CellChat (v1.6.1) (Jin et al., 2021). Communication probability, pathway-level information flow, and signaling centrality were calculated for control, nicotine, and smoke conditions separately. Inferred ligand-receptor interactions and signaling patterns were visualized using hierarchical, chord, and circle plots. For condition-specific analyses, shared cluster identities and cell-type annotations were retained across conditions. Because one pooled library was generated per condition, between-condition differences in CellChat-derived network metrics were interpreted descriptively rather than as replicate-based statistical differences; differences in nuclei numbers across clusters and conditions were also considered when interpreting network characteristics.

## Results

### Single-nucleus transcriptomic profiling maps major brain cell types across whole-brain samples under nicotine and smoke exposure

To determine a nicotine administration dose that produced systemic nicotine exposure comparable to cigarette smoke, we first performed a preliminary dose-comparison experiment. Mice received daily subcutaneous injections of nicotine at 1, 2, or 3 mg/kg for 14 consecutive days, while a separate group was exposed to mainstream cigarette smoke at 1000 mg/m^3^. Serum samples were collected 1 h after the final exposure for measurement of nicotine and cotinine concentrations. Serum nicotine concentrations following exposure to 1,000 mg/m^3^ cigarette smoke were comparable to those observed following administration of 1 mg/kg nicotine (Table 1). Therefore, 1 mg/kg nicotine was selected as the pharmacological exposure dose for the subsequent snRNA-seq experiments.

**Table 1:** Serum nicotine concentrations following cigarette smoke exposure or nicotine injection.

Treatment	Serum cotinine contents (ng/ml)
Air exposure	2.37 ± 0.78
1,000 mg/m^3^ cigarette smoke	17.20 ± 4.85
Vehicle subcutaneous injection	2.04 ± 0.23
1 mg/kg nicotine subcutaneous injection	16.28 ± 3.06
2 mg/kg nicotine subcutaneous injection	36.84 ± 14.60
3 mg/kg nicotine subcutaneous injection	51.52 ± 15.39

To characterize the cellular landscape and transcriptional responses of the brain to nicotine and smoke exposure, we performed snRNA-seq on whole-brain samples from adult C57BL/6 mice subjected to two treatment protocols. For the snRNA-seq experiment, mice in the pharmacological exposure group received daily subcutaneous injections of nicotine at 1 mg/kg for 14 consecutive days, whereas mice in the environmental exposure group were exposed to whole-body mainstream cigarette smoke at 1000 mg/m^3^ for 2 h daily for 14 consecutive days. For both protocols, brain tissues were harvested 1 h after the final exposure and processed for snRNA-seq (Figure 1A). After quality control and joint analysis, we retained a total of 26,752 high-quality nuclei, including 10,585 from control, 6,779 from nicotine-treated, and 9,388 from smoke-exposed mice. Dimensionality reduction and unsupervised clustering identified 18 transcriptionally distinct clusters represented across all three conditions (Figures 1B,C). Cell-type annotation of the whole-brain dataset was performed at the broad cell-class level using canonical marker gene expression (Supplementary Figure S1A). These markers identify major neuronal and glial classes rather than region-specific subtypes. Cluster 0, which represented the largest population across groups, expressed high levels of *Slc17a7*, and multiple clusters (e.g., 3, 10, 15) expressed *Slc17a7* and *Satb2*, consistent with excitatory neurons (Figure 1D; Supplementary Figure S1B). Astrocytes (Cluster 13, 14, 16) were identified by expression of *Slc4a4* and *Slc1a3* (Figure 1E), while microglia (Cluster 4, 19 17) expressed *Fyb* and *Ctss* (Figure 1F). Clusters 2, 5, 7, 8 and 12 expressed *Gad1*, *Gad2*, and *Lamp5*, corresponding to inhibitory neuronal subtypes (Figure 1G), whereas clusters 1 and 6 expressed *Mog* and *Opalin*, indicating oligodendrocytes (Figure 1H). Together, these data establish a single-nucleus transcriptomic map of the adult mouse brain following nicotine and smoke exposure, with identification of major cell classes and transcriptionally distinct clusters across experimental conditions.

**Figure 1:**
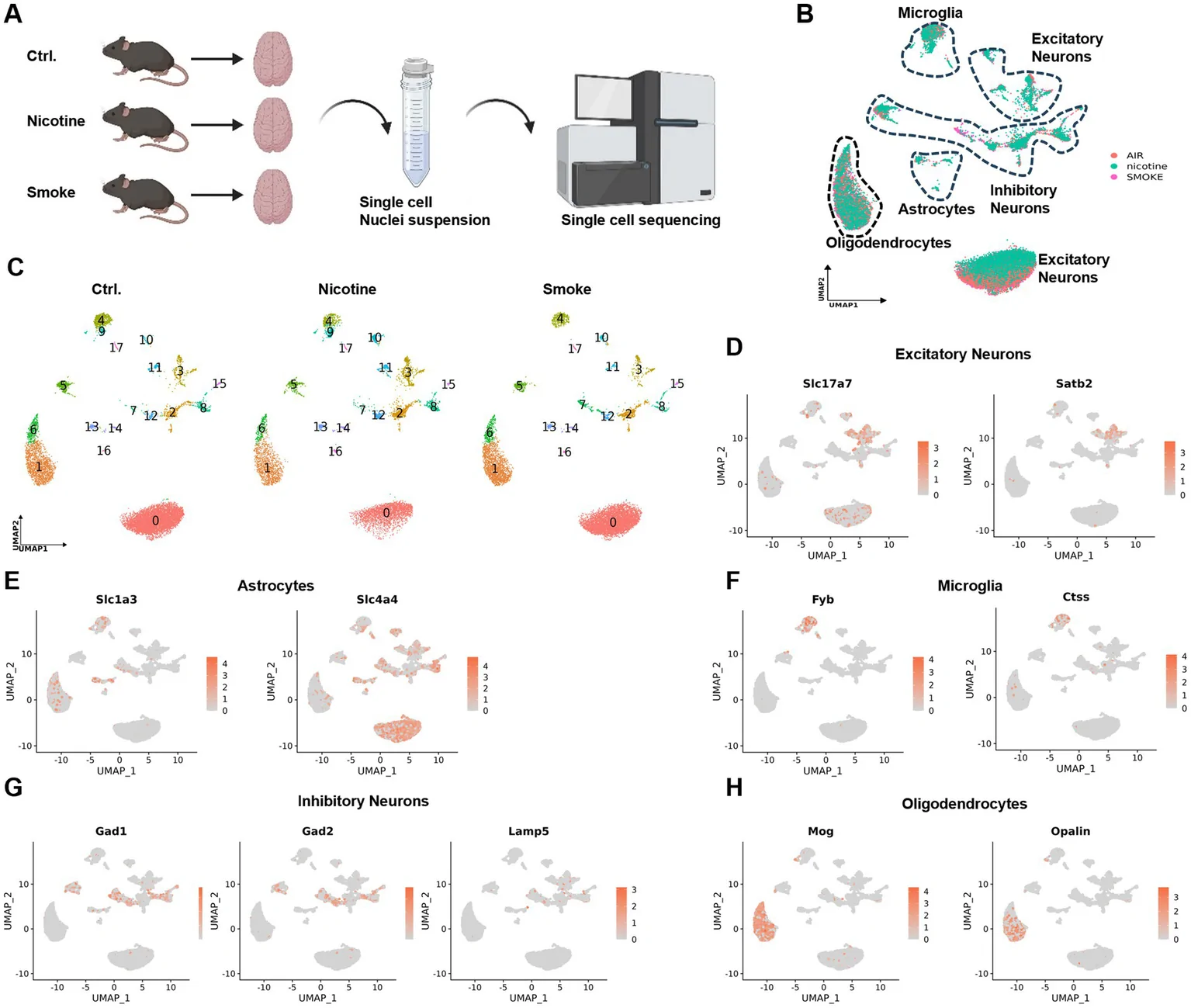
Single-nucleus transcriptomic profiling of adult mouse whole-brain samples following nicotine and smoke exposure. **(A)** Experimental timeline and schematic for snRNA-seq. **(B)** Joint UMAP embedding demonstrating distinct transcriptional clusters across control, nicotine, and smoke conditions. **(C)** UMAP visualization of jointly analyzed snRNA-seq data, identifying 18 distinct cell clusters across control, nicotine-treated, and smoke-exposed samples. **(D–H)** Expression of canonical markers used for cell type annotation: **(D)** Excitatory neurons (Slc17a7, Satb2); **(E)** Astrocytes (Slc4a4, Slc1a3); **(F)** Microglia (Fyb, Ctss); **(G)** Inhibitory neurons (Gad1, Gad2, Lamp5); **(H)** Oligodendrocytes (Mog, Opalin).

### Nicotine and smoke exposure are associated with distinct inferred intercellular communication and NRXN-related patterns

To characterize treatment-associated differences in brain-wide intercellular communication, we applied CellChat to infer ligand-receptor communication patterns across control, nicotine, and smoke conditions. Analysis of global interaction strength showed differences in inferred incoming and outgoing communication across multiple clusters, including clusters 0, 1, 2, and 3. Cluster 0 (excitatory neurons) exhibited relatively high inferred outgoing communication across all three conditions and was consistently predicted to act as a major signaling source (Figure 2A). At the pathway level, nicotine exposure was associated with higher inferred relative and absolute information flow for several pathways, including NRXN, NCAM, and NRG (Neuregulin) (Figure 2B, left panel). Smoke exposure showed a distinct inferred communication profile involving extracellular matrix and immune-related pathways, together with lower inferred NRXN information flow (Figure 2B, right panel). Communication heatmaps further indicated that, under nicotine exposure, cluster 0 was the predominant predicted NRXN sender, with cluster 1 (oligodendrocytes) and cluster 3 (excitatory neurons) as major predicted receivers (Figure 2C, left panel). Under smoke exposure, cluster 0 was likewise predicted as a major NRXN sender to cluster 1 (oligodendrocytes) (Figure 2C, right panel). Together, these results indicate condition-associated differences in inferred NRXN-related communication patterns. Because CellChat infers ligand-receptor communication from transcriptomic data, these predictions do not establish functional signaling and require independent experimental validation.

**Figure 2:**
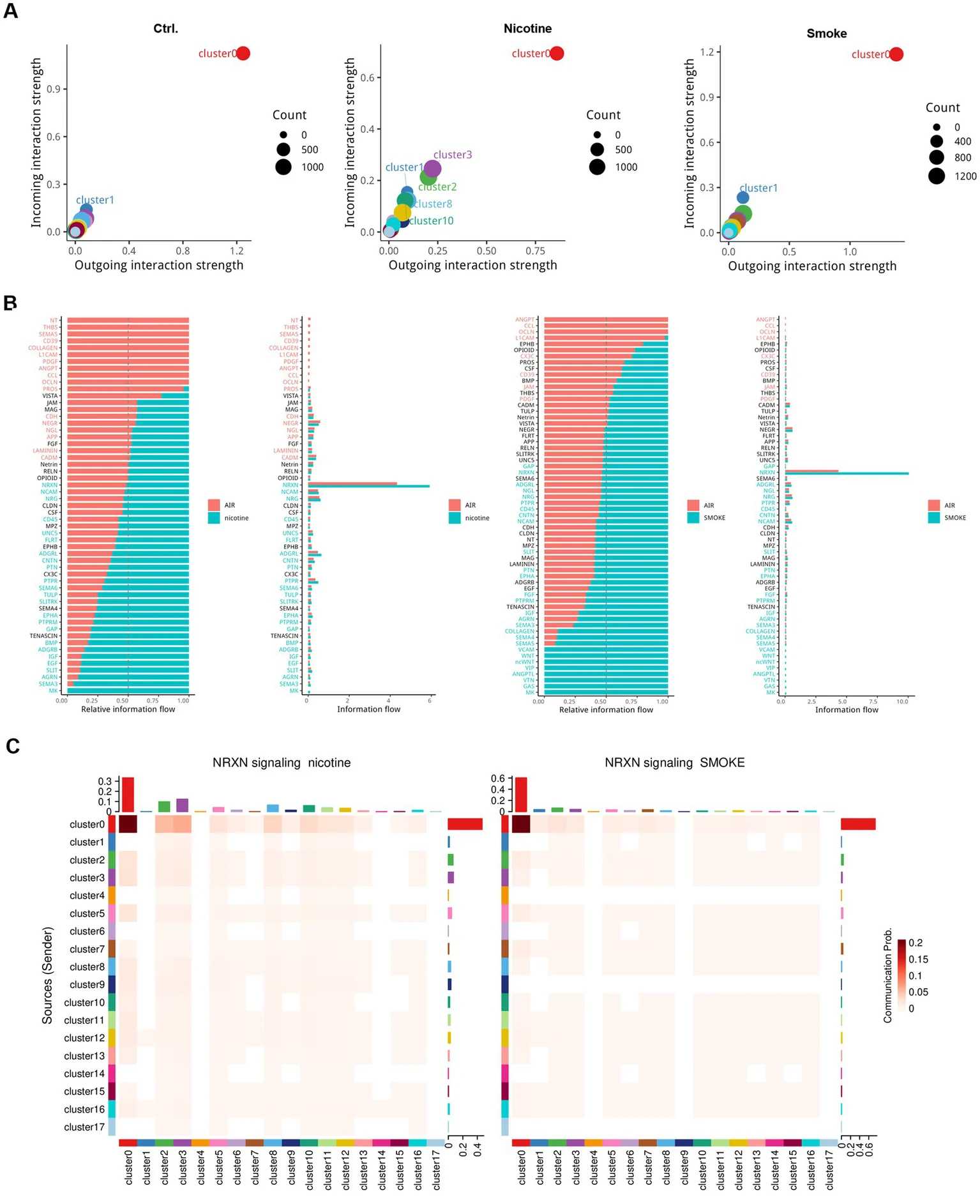
Nicotine and smoke exposure are associated with distinct CellChat-inferred brain-wide communication patterns. **(A)** CellChat-inferred incoming and outgoing interaction strengths across clusters under control, nicotine, and smoke conditions; identical axis limits and a common point-size scale were used across panels to facilitate direct visual comparison. **(B)** Relative information flow analysis showing condition-associated differences in inferred signaling pathways, including NRXN, PTN, and NRG under nicotine exposure. **(C)** Heatmaps depicting predicted NRXN source-target relationships, with excitatory neurons (cluster 0) predicted as the major sender to oligodendrocytes (cluster 1) and other excitatory neurons (cluster 3) under nicotine exposure; under smoke exposure, cluster 0 was also predicted to signal to oligodendrocytes.

### Oligodendrocyte-specific transcriptional responses and PISD induction following tobacco smoke exposure

To investigate the transcriptional alterations induced by nicotine and cigarette smoke exposure, we performed DGE analysis across major brain cell types. A bar plot summarizing the number of significantly upregulated and downregulated genes (adjusted *p* < 0.05, |log₂FC| > 0.25) revealed that inhibitory neurons and oligodendrocytes exhibited the most pronounced transcriptional responses under both nicotine and smoke conditions (Figure 3A). Notably, smoke exposure led to a substantial increase in the number of upregulated genes in oligodendrocytes, indicating a cell type-specific transcriptional response to smoke exposure. To further characterize the biological pathways enriched in response to smoke, KEGG pathway enrichment analysis was conducted using the DEGs from the smoke-treated group compared to controls. The top enriched pathways included thyroid hormone synthesis, oxytocin signaling, cAMP-PKG signaling, Wnt signaling, and platelet activation (Figure 3B). These results indicate enrichment of genes assigned to neuroendocrine and metabolism-related pathways following smoke exposure and do not by themselves demonstrate functional pathway activation. Importantly, a volcano plot of DEGs in oligodendrocytes revealed marked upregulation of PISD (*phosphatidylserine decarboxylase*) (Figure 3C). PISD encodes a mitochondrial enzyme responsible for converting phosphatidylserine to phosphatidylethanolamine (PE), a key phospholipid essential for mitochondrial membrane integrity and myelin structure. The observed increase in PISD expression represents a transcriptomic signature potentially associated with mitochondrial phospholipid metabolism. However, mitochondrial function, lipid composition, and stress adaptation were not directly assessed in this study, and the functional significance of PISD upregulation therefore requires further experimental validation. In the nicotine-treated group, the most prominent transcriptional changes were observed in microglia. To explore how microglia respond to nicotine stimulation, we examined clusters 4 and 9, which were annotated as microglia based on canonical marker expression (Supplementary Figure S2A). KEGG enrichment analysis identified transcriptomic signatures associated with glutamatergic synapse, dopaminergic synapse, cAMP signaling, long-term potentiation, and addiction-related pathways. These enrichment patterns suggest potential involvement of neuronal and synaptic signaling programs but do not by themselves demonstrate functional pathway activation (Supplementary Figure S2A). The volcano plot showed increased expression of neuronal signaling-associated genes (e.g., Nrxn1 and Nrxn3). Together, these changes indicate a neuronal and synaptic-signaling-associated transcriptional response rather than evidence of an anti-plasticity state (Supplementary Figure S2B).

**Figure 3:**
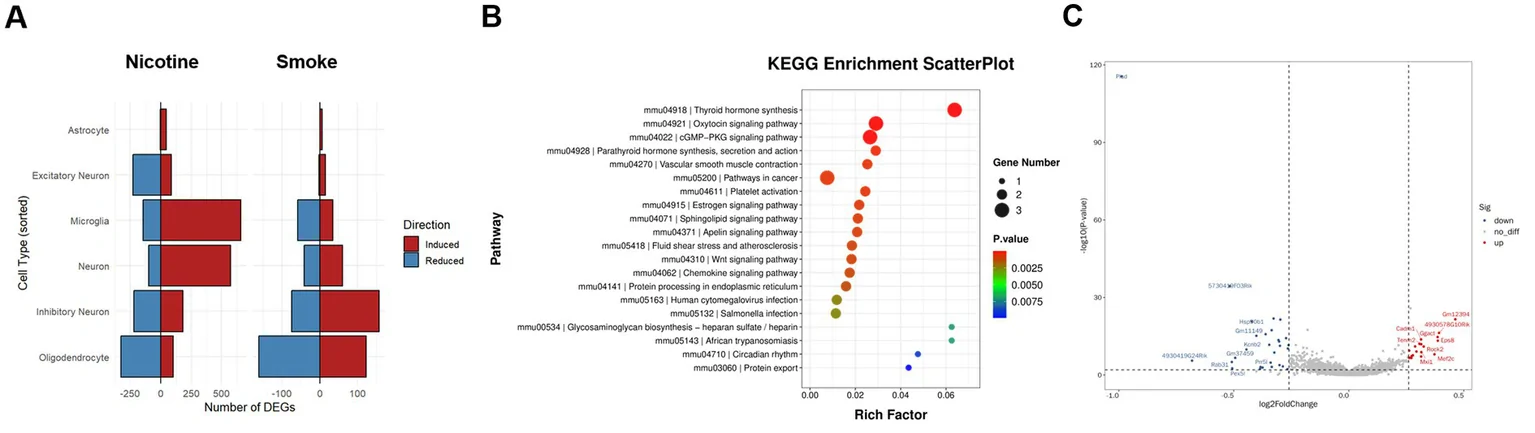
Oligodendrocyte-specific transcriptional responses following nicotine and smoke exposure. **(A)** Bar plot summarizing the number of significantly upregulated and downregulated genes across brain cell types under nicotine and smoke conditions, highlighting pronounced responses in inhibitory neurons and oligodendrocytes. **(B)** KEGG pathway enrichment analysis using clusterProfiler (v4.4.4) (Kanehisa and Goto, 2000; Kanehisa et al., 2025) for smoke-exposed oligodendrocytes, showing enrichment of neuroendocrine and metabolism-related pathways including thyroid hormone synthesis, oxytocin signaling, cAMP-PKG, and Wnt signaling pathways. **(C)** Volcano plot highlighting increased PISD expression in oligodendrocytes following smoke exposure.

### Single-nucleus RNA-seq of the mouse VTA reveals diverse neuronal and glial populations across control, nicotine, and smoke exposure

Given the pivotal role of the VTA in addiction-related circuitry, we sought to investigate the cellular and molecular impact of nicotine and tobacco smoke exposure specifically within this region. To this end, we microdissected the VTA from adult mouse brain sections following three treatment conditions: control (Ctrl), nicotine, and cigarette smoke exposure, and performed snRNA-seq (Figure 4A). High-quality single-nucleus suspensions were obtained and processed for library preparation and sequencing. Following quality control, dimensionality reduction, and joint analysis, we constructed a comprehensive single-nucleus transcriptomic map of the VTA, encompassing a total of 29,775 nuclei. Specifically, 13,659 nuclei were derived from the control group, 4,530 from the nicotine group, and 11,586 from the smoke exposure group (Figure 4B). UMAP embedding and unsupervised clustering revealed 27 transcriptionally distinct clusters that were largely conserved across conditions, indicating a high degree of cellular heterogeneity in the VTA. While the overall cluster composition was similar between groups, descriptive differences in cell-type proportions were observed between Ctrl, nicotine, and smoke-treated samples (Figure 4C; Supplementary Figure S3). Because independent animal-level biological replicates were not available, these differences were not interpreted as evidence of treatment-induced changes in cell abundance. To annotate cluster identities, we used canonical marker genes for broad neuronal and glial cell classes (Supplementary Figure S3). Major cell types identified included excitatory neurons (*Slc17a7*, *Satb2*), inhibitory neurons (*Gad1*, *Gad2*), oligodendrocyte precursor cells (OPCs; *Pdgfra*), mature oligodendrocytes (*Mog*, *Opalin*), astrocytes (*Slc4a4*, *Slc1a3*), and microglia (*Fyb*, *Ctss*) (Figures 4D–I). These marker expression patterns supported broad annotation of neuronal and glial lineages. The joint UMAP projection showed transcriptional separation of excitatory and inhibitory neuronal populations, while glial populations occupied distinct regions of UMAP space. Notably, all major cell types were represented in each treatment condition, indicating successful isolation of nuclei from the VTA and robust recovery of cellular diversity. Collectively, these data establish a high-resolution single-nucleus transcriptomic map of the VTA and provide a foundational framework for dissecting the cell-type-specific effects of nicotine and cigarette smoke exposure.

**Figure 4:**
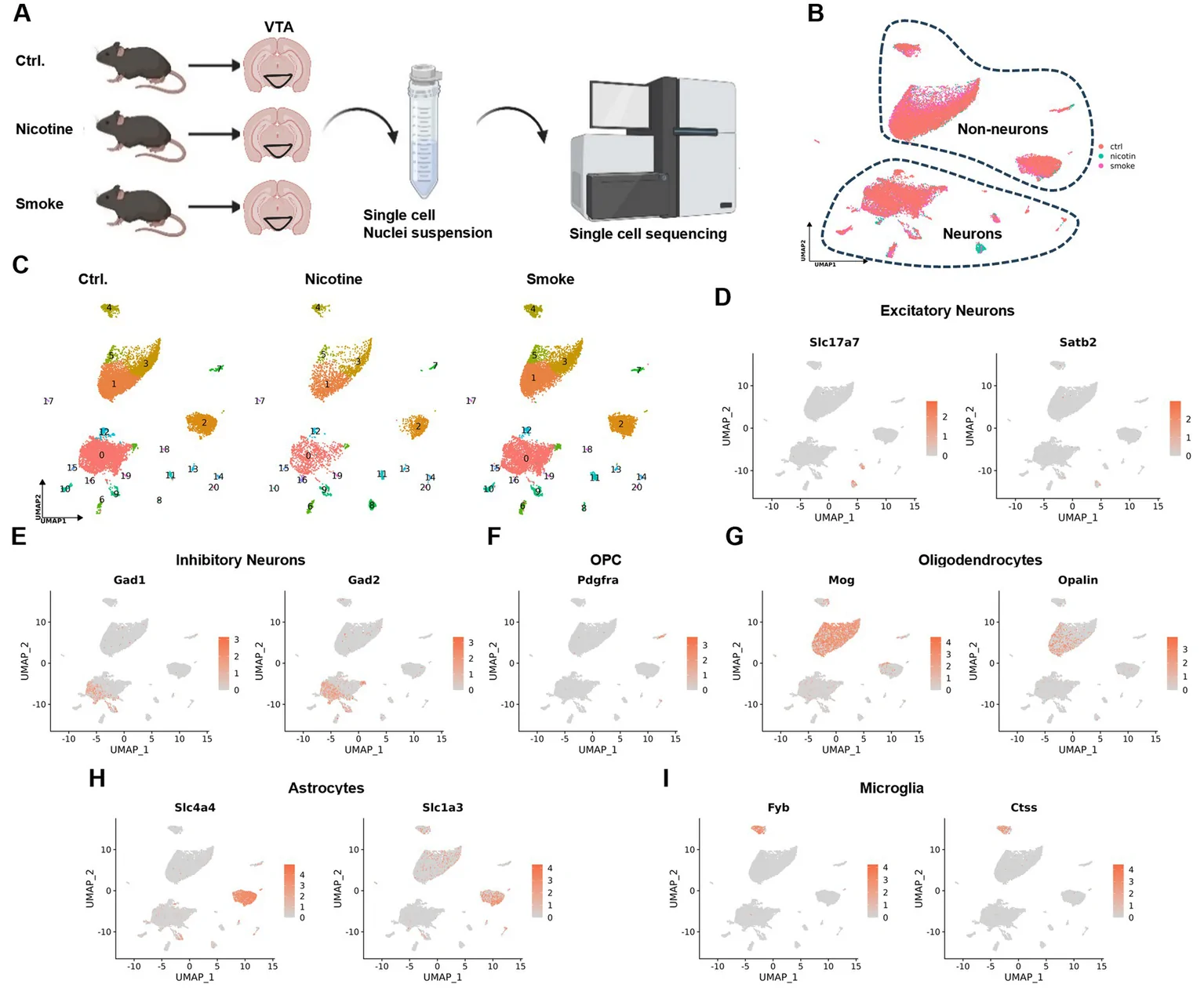
Single-nucleus RNA sequencing identifies diverse neuronal and glial populations within the mouse VTA following nicotine and smoke exposure. **(A)** Schematic of experimental design for snRNA-seq on microdissected VTA tissues. **(B)** Joint UMAP embedding demonstrating distinct transcriptional clusters across control, nicotine, and smoke conditions. **(C)** Unsupervised clustering revealed 27 transcriptionally distinct clusters. **(D–I)** Dot plot visualization of canonical marker genes used for annotation of major cell types: **(D)** excitatory neurons (Slc17a7, Satb2), **(E)** inhibitory neurons (Gad1, Gad2), **(F)** oligodendrocyte precursor cells (Pdgfra), **(G)** mature oligodendrocytes (Mog, Opalin), **(H)** astrocytes (Slc4a4, Slc1a3), and **(I)** microglia (Fyb, Ctss).

### Characterization of VTA neuronal subclusters based on canonical neurotransmitter marker profiles

To further resolve the cellular heterogeneity within the VTA neuronal population, we reclustered neuronal cells and identified 17 transcriptionally distinct subclusters (Figure 5A). These subclusters showed separation in UMAP space, reflecting transcriptional differences among neuronal populations. To classify neurotransmitter identities, we evaluated canonical marker genes for GABAergic (e.g., *Slc6a1, Gad1, Gad2*), glutamatergic (e.g., *Slc17a6, Slc17a7*), and dopaminergic neurons (e.g., *Th, Ddc, Slc6a3, Slc18a2*). Dot plot visualization revealed diverse expression patterns across clusters, supporting assignment of broad neurotransmitter-associated marker profiles (Figure 5B). For example, clusters 4 and 14 exhibited high expression of *Th* and *Slc6a3*, consistent with a dopaminergic (DA) identity. Clusters 3 and 7 expressed *Slc17a6* with low GABAergic gene expression, suggesting glutamatergic (GLU) profiles, while clusters 0, 1, 2, and 9 displayed predominant GABAergic gene expression. To complement this, we performed binary co-expression analysis of key neurotransmitter genes within each cluster (expression threshold > 0.1). Co-expression frequency heatmaps revealed distinct gene modules consistent with neurotransmitter assignment (Figure 5C). Clusters 5, 6, 10, 13, and 16 showed strong co-expression among GABAergic genes (*Gad1, Gad2, Slc32a1*), whereas clusters 3, 7, and 8 featured co-expression of glutamatergic markers. Notably, clusters 11 and 15 showed co-expression of both GABAergic and glutamatergic marker genes, indicating mixed neurotransmitter-associated transcriptional profiles; this co-expression alone was not considered sufficient to establish bona fide dual-transmitter identity (Figure 5C). These results collectively indicate transcriptional heterogeneity among VTA neuronal subpopulations, including clusters with predominant GABAergic, glutamatergic, or dopaminergic marker profiles and clusters with mixed marker expression.

**Figure 5:**
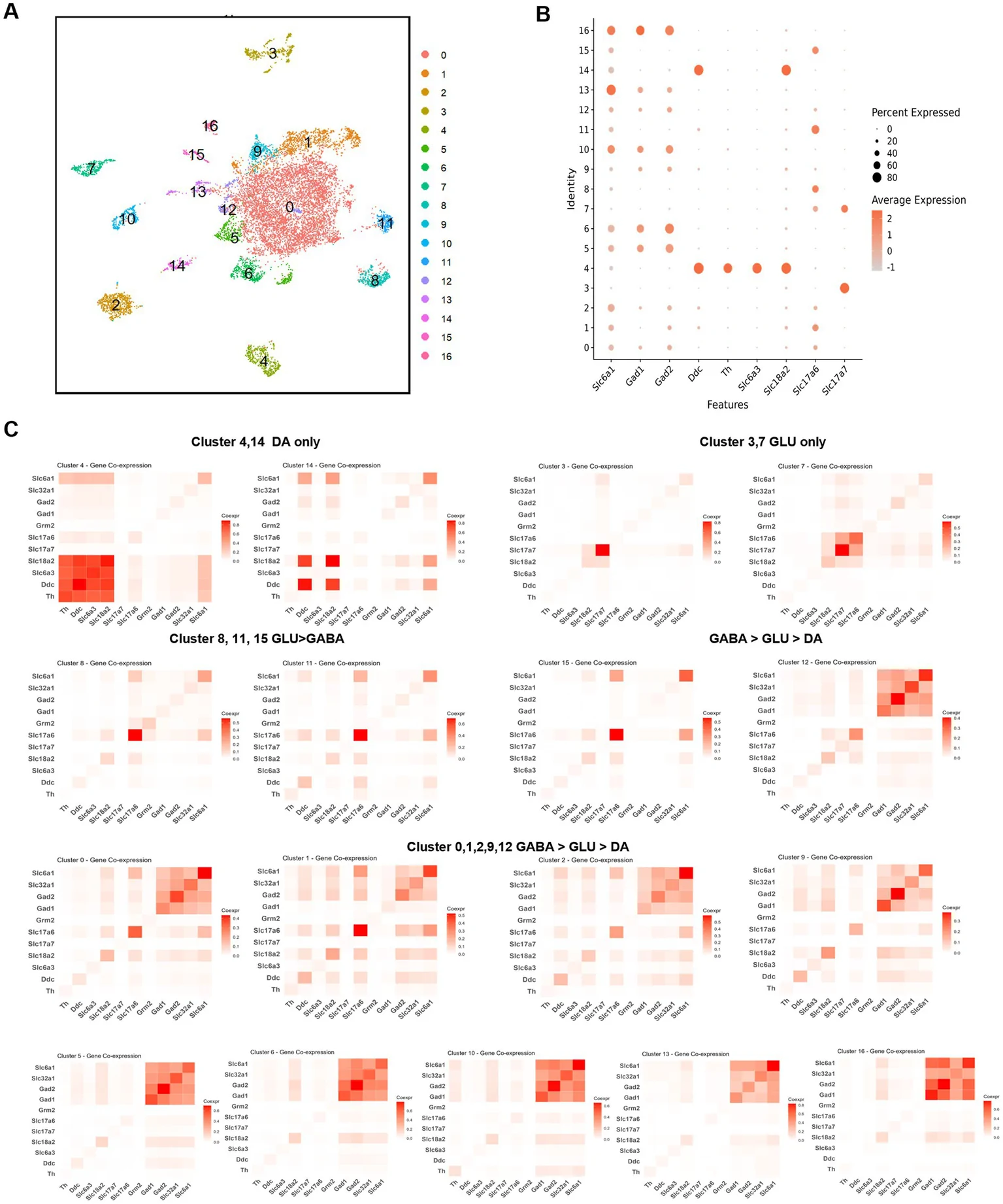
Characterization of VTA neuronal subpopulations based on neurotransmitter gene expression profiles. **(A)** Reclustering of neuronal cells identified 17 transcriptionally distinct neuronal subclusters. **(B,C)** Dot plots and heatmaps illustrating expression and co-expression patterns of canonical neurotransmitter markers for GABAergic (Slc6a1, Gad1, Gad2), glutamatergic (Slc17a6, Slc17a7), and dopaminergic neurons (Th, Ddc, Slc6a3, Slc18a2), supporting assignment of neurotransmitter-associated marker profiles, including clusters with mixed GABAergic and glutamatergic marker expression.

### Distinct transcriptomic signatures associated with nicotine and smoke exposure in VTA neurons

To investigate the transcriptomic alterations induced by nicotine and smoke exposure, we performed DEG analysis across distinct VTA neuronal populations, followed by KEGG pathway enrichment analysis. As shown in (Figure 6A), both nicotine and smoke exposure were associated with widespread transcriptional changes across multiple neuronal subtypes. Notably, cluster 0-annotated as a mixed neuronal population dominated by GABAergic neurons, with smaller subsets of glutamatergic and dopaminergic neurons-exhibited the most substantial transcriptional alterations, with over 1,000 DEGs identified in response to either treatment. Cluster 0 therefore showed the largest number of detected DEGs among the analyzed neuronal clusters, although DEG counts alone do not establish increased biological susceptibility. Additional clusters, including 4, 5, and 11, also showed moderate numbers of DEGs following nicotine exposure. The distribution and number of detected DEGs differed across neuronal clusters between nicotine and smoke exposure. KEGG pathway enrichment analysis of DEGs following nicotine exposure revealed distinct biological processes and signaling pathways (Figure 6B). Enriched pathways include lysosome, phagosome, ferroptosis, Hippo, and cAMP signaling, as well as several metabolic processes. Notably, oxidative phosphorylation (OXPHOS) and other energy-related pathways were prominently enriched, indicating transcriptional enrichment of genes associated with cellular metabolism rather than direct evidence of altered mitochondrial function. In comparison, DEGs induced by smoke exposure showed a different distribution of enriched pathway annotations (Figure 6B). Enriched pathways included environmental information processing (e.g., MAPK, calcium, PI3K-Akt signaling), genetic information processing (e.g., ribosome biogenesis, protein processing in the endoplasmic reticulum), pathways associated with human diseases (e.g., neurodegeneration), and organismal systems such as circadian entrainment and dopaminergic synapse. These enrichments reflect transcriptional representation of genes assigned to these KEGG categories and do not establish neurodegeneration, altered circadian behavior, or functional dopaminergic synaptic changes. Metabolic and OXPHOS-related pathways were enriched under both nicotine and smoke exposure, indicating shared transcriptional signatures associated with mitochondrial and energy-metabolism-related processes. These findings do not establish functional metabolic reprogramming and require further validation. Collectively, these results delineate shared and distinct transcriptomic responses to nicotine and smoke exposure in VTA neurons and identify pathway-associated transcriptional signatures that may be relevant to exposure-associated neuronal responses.

**Figure 6:**
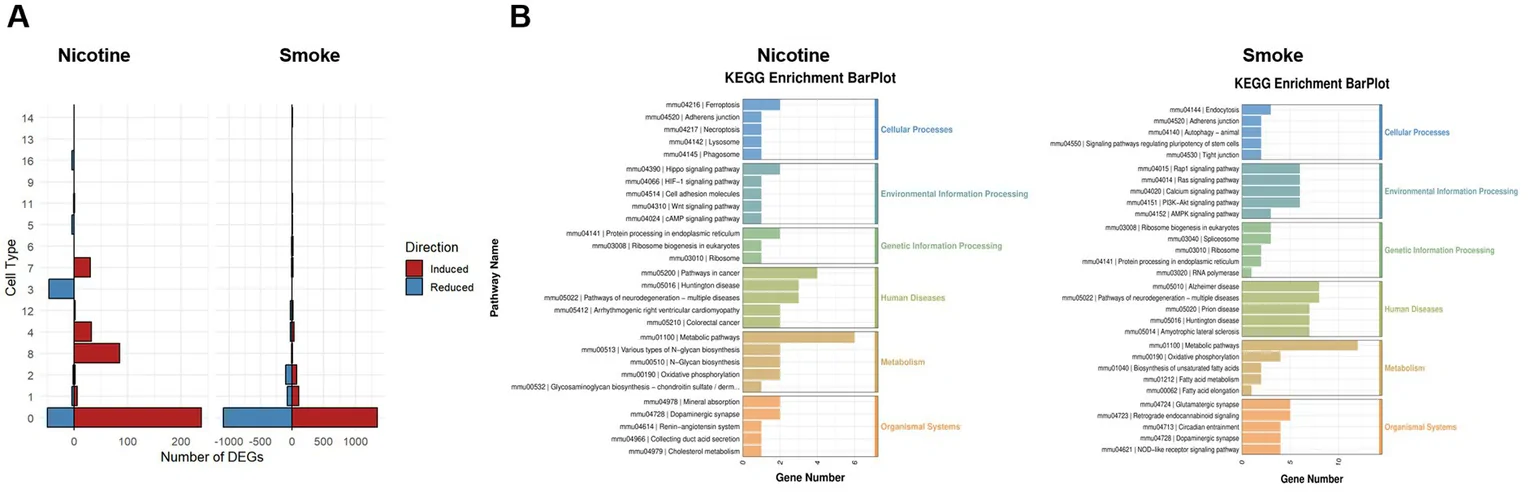
Differential transcriptomic responses of VTA neuronal clusters to nicotine and smoke exposure. **(A)** Barplot of DEG counts per neuronal cluster, indicating pronounced transcriptional changes particularly in cluster 0 under both nicotine and smoke conditions. **(B)** KEGG pathway enrichment analysis for nicotine (left panel) and smoke (right panel) conditions, showing enrichment of genes annotated to metabolic, lysosome, ferroptosis, and signaling pathways such as MAPK and PI3K-Akt, describing treatment-associated transcriptional signatures rather than direct functional pathway activation.

### Inferred intercellular communication patterns in VTA neurons under nicotine and smoke exposure

To characterize treatment-associated differences in predicted cell–cell communication in the VTA, we applied CellChat-based network analysis to the control, nicotine, and smoke conditions. As shown in Figure 7A, the global inferred communication network displayed condition-associated differences in incoming and outgoing interaction strength across neuronal clusters. Cluster 0 showed relatively high inferred outgoing communication in both nicotine and smoke-exposed samples, accompanied by relatively high total interaction counts. In nicotine-exposed samples, clusters 3 and 4 also showed relatively increased inferred interaction strength, whereas smoke exposure showed relatively greater inferred outgoing communication involving cluster 1. These patterns describe differences in the inferred communication network across conditions and do not by themselves indicate functional activation or identify specific regulatory roles of individual clusters.

**Figure 7:**
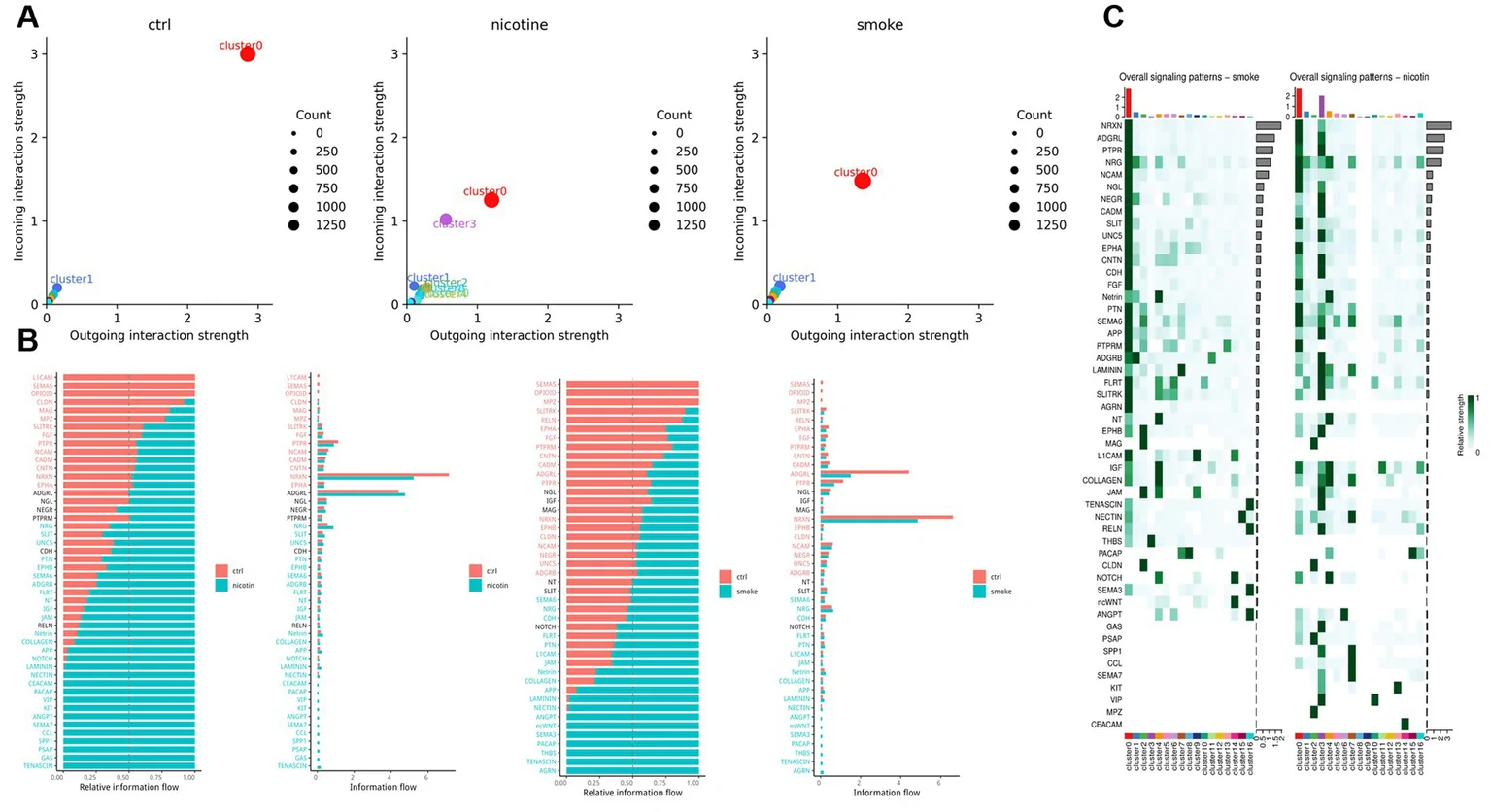
CellChat-inferred communication patterns in VTA neurons under nicotine and smoke exposure. **(A)** Global interaction strength visualizations showing condition-associated differences in inferred outgoing and incoming communication, including relatively high inferred outgoing communication from cluster 0 in the nicotine and smoke datasets. **(B)** Signaling pathway-specific relative information flow analysis comparing control, nicotine, and smoke conditions, showing differences in inferred information flow for pathways including NRXN, ADGRE, WNT, and IL6. **(C)** Heatmap overview displaying predicted ligand-receptor communication probabilities across conditions, emphasizing distinct and partially overlapping inferred communication patterns.

Comparative analysis of pathway-level relative information flow revealed condition-associated differences across signaling pathways (Figure 7B). Nicotine exposure showed relatively greater inferred information flow for several pathways, including NRXN and ADGRE, whereas control samples showed relatively greater information flow for pathways such as SEMA3 and EPHB. Smoke exposure showed relatively greater inferred information flow for several pathways compared with control, particularly WNT-, MIF-, ANGPTL-, and IL6-related communication. The heatmap further demonstrated distinct but partially overlapping predicted ligand-receptor communication patterns across the three conditions (Figure 7C). NRG-, NECTIN-, and NOTCH-related pathways were among the communication pathways detected across the exposure conditions, but these pathway categories represent broad intercellular signaling systems and should not be interpreted as direct evidence of dopaminergic synaptic signaling.

Overall, the CellChat analysis revealed distinct patterns of inferred intercellular communication associated with nicotine and smoke exposure in the VTA. Because the analysis was based on pooled samples without independent animal-level biological replicates, these findings are considered descriptive and exploratory and do not establish statistically validated changes in functional cell–cell signaling.

## Discussion

Our study provides a comprehensive single-nucleus transcriptomic atlas of adult mouse brains exposed to nicotine and cigarette smoke, providing a detailed view of cell-type-specific transcriptional responses and predicted intercellular communication changes. This dataset provides a single-nucleus transcriptomic resource for examining VTA cellular responses to smoke exposure. Our analyses identify distinct transcriptomic signatures and predicted communication patterns across nicotine and smoke conditions, highlighting exposure-associated molecular differences.

In our study, single-nucleus transcriptomic analysis combined with CellChat identified NRXN as one of the pathways showing prominent changes in inferred whole-brain intercellular communication following nicotine or tobacco smoke exposure. This observation suggests that NRXN-related communication may be involved in exposure-associated changes in predicted intercellular signaling. Neurexins and their postsynaptic partners, neuroligins, are essential synaptic cell-adhesion molecules that mediate trans-synaptic communication. These molecules are crucial for the organization, specificity, and stability of excitatory and inhibitory synapses, and they play fundamental roles in synaptic efficacy and plasticity, which are key processes in learning, memory, and reward-associated behaviors (Südhof, 2008; Südhof, 2017; Kasem et al., 2018). Among the neurexin gene family, NRXN3 has emerged as a particularly relevant candidate in the context of substance dependence. Multiple GWAS and candidate gene studies have reported associations between NRXN3 polymorphisms and nicotine, alcohol, and cocaine dependence, as well as addiction-related traits such as impulsivity and reward sensitivity. Taken together, these prior genetic associations provide context for the NRXN-related transcriptional changes observed in the present study but do not establish a causal role for NRXN signaling in exposure-associated phenotypes (Novak et al., 2009; Docampo et al., 2012; Kelai et al., 2008). Changes in inferred NRXN-related communication were observed under both exposure conditions, suggesting a potentially shared transcriptomic feature; however, CellChat inference alone does not establish functional pathway activation or a convergent mechanism of synaptic adaptation. Further studies are warranted to dissect the causal role of NRXN3 in mediating addictive behaviors and to explore its potential as a therapeutic target.

The VTA plays a central role in the neurocircuitry of addiction, acting as a critical hub in the mesolimbic dopamine pathway that mediates reward, reinforcement, and motivational processes. VTA neurons, particularly dopaminergic, GABAergic, and glutamatergic subtypes, project broadly to limbic and cortical regions and are dynamically regulated by addictive substances (Yoo et al., 2016; Qi et al., 2016; Cai and Tong, 2022; Prévost et al., 2025; Beier et al., 2015). While the effects of nicotine on the VTA have been well-documented in previous studies—demonstrating its ability to activate dopaminergic neurons, enhance dopamine release, and modulate synaptic plasticity within this region -the impact of whole cigarette smoke exposure on the VTA at single-nucleus resolution remains largely unexplored. This represents a significant knowledge gap, as cigarette smoke is a complex mixture of thousands of chemicals, many of which may exert effects distinct from pure nicotine (Chen et al., 2022). In this context, our study provides a novel contribution by comprehensively characterizing the cellular composition and transcriptional dynamics of the VTA under both nicotine and smoke exposure using single-nucleus RNA sequencing. Building on recent efforts to map the VTA in animal models-such as the study *An atlas of transcriptionally defined cell populations in the rat ventral tegmental area*, which defined diverse cell populations based on transcriptional signatures-we were able to annotate 27 transcriptionally distinct clusters in the mouse VTA. This detailed cellular map enables high-resolution examination of exposure-associated transcriptional responses across neuronal subtypes.

Importantly, our data align with and extend findings from prior reports such as *Ventral tegmental area glutamate neurons co-release GABA and promote positive reinforcement*, which revealed the functional complexity and heterogeneity of VTA glutamatergic neurons. We observed distinct transcriptional profiles following nicotine and smoke exposure, with nicotine associated with enrichment of synaptic and addiction-related pathway annotations and smoke showing broader enrichment of immune and metabolism-associated transcripts and pathways. This direct comparison of nicotine and cigarette smoke exposure at single-nucleus transcriptomic resolution revealed overlapping and divergent patterns of exposure-associated neuronal transcriptional responses. These findings provide a transcriptomic framework for comparing nicotine and whole-smoke exposure in the VTA and highlight the importance of considering the complexity of whole-smoke exposure.

Nevertheless, the present study has important limitations. Because the study is based primarily on transcriptomic profiling and computational pathway analysis, the inferred biological processes should be interpreted as hypotheses or transcriptomic signatures rather than direct evidence of functional pathway activation. In particular, the proposed associations between PISD expression and mitochondrial lipid metabolism, as well as the enrichment of synaptic and addiction-related pathways, require validation using complementary biochemical, imaging, electrophysiological, or other functional approaches. In addition, tissues from three animals within each condition were pooled before nuclei isolation, so independent animal-level biological replicates were unavailable for replicate-based differential-expression or CellChat comparisons. CellChat findings should therefore be interpreted as descriptive, transcriptome-based predictions of potential ligand–receptor communication rather than direct evidence of functional signaling.

## Data Availability

The datasets generated and/or analyzed during the current study are available in the GEO repository (https://www.ncbi.nlm.nih.gov/geo/query/acc.cgi?acc=GSE336012).

## References

[ref1] Bariselli S. Glangetas C. Tzanoulinou S. Bellone C. (2016). Ventral tegmental area subcircuits process rewarding and aversive experiences. J. Neurochem. 139, 1071–1080. doi: 10.1111/jnc.13779, 27546491

[ref2] Beier K. T. Steinberg E. E. DeLoach K. E. Xie S. Miyamichi K. Schwarz L. . (2015). Circuit architecture of VTA dopamine neurons revealed by systematic input-output mapping. Cell 162, 622–634. doi: 10.1016/j.cell.2015.07.015, 26232228 PMC4522312

[ref3] Benowitz N. L. (2008). Neurobiology of nicotine addiction: implications for smoking cessation treatment. Am. J. Med. 121, S3–S10. doi: 10.1016/j.amjmed.2008.01.015, 18342164

[ref4] Bierut L. J. Madden P. A. F. Breslau N. Johnson E. O. Hatsukami D. Pomerleau O. F. . (2007). Novel genes identified in a high-density genome wide association study for nicotine dependence. Hum. Mol. Genet. 16, 24–35. doi: 10.1093/hmg/ddl441, 17158188 PMC2278047

[ref5] Cai J. Tong Q. (2022). Anatomy and function of ventral tegmental area glutamate neurons. Front. Neural Circuits 16:867053. doi: 10.3389/fncir.2022.867053, 35669454 PMC9164627

[ref6] Chen B. Sun L. Zeng G. Shen Z. Wang K. Yin L. . (2022). Gut bacteria alleviate smoking-related NASH by degrading gut nicotine. Nature 610, 562–568. doi: 10.1038/s41586-022-05299-4, 36261549 PMC9589931

[ref7] Docampo E. Ribasés M. Gratacòs M. Bruguera E. Cabezas C. Sánchez-Mora C. . (2012). Association of neurexin 3 polymorphisms with smoking behavior. Genes Brain Behav. 11, 704–711. doi: 10.1111/j.1601-183X.2012.00815.x, 22716474

[ref9008] Hao Y. Hao S. Andersen-Nissen E. Mauck W. M. 3rd Zheng S. Butler A. . (2021). Integrated analysis of multimodal single-cell data. Cell 184, 3573–3587.e29. doi: 10.1016/j.cell.2021.04.048 PMC8238499 34062119

[ref9] Jin S. Guerrero-Juarez C. F. Zhang L. Chang I. Ramos R. Kuan C. H. . (2021). Inference and analysis of cell-cell communication using CellChat. Nat. Commun. 12:1088. doi: 10.1038/s41467-021-21246-9, 33597522 PMC7889871

[ref10] Kanehisa M. Furumichi M. Sato Y. Matsuura Y. Ishiguro-Watanabe M. (2025). KEGG: biological systems database as a model of the real world. Nucleic Acids Res. 53, D672–D677. doi: 10.1093/nar/gkae909, 39417505 PMC11701520

[ref11] Kanehisa M. Goto S. (2000). KEGG: Kyoto encyclopedia of genes and genomes. Nucleic Acids Res. 28, 27–30. doi: 10.1093/nar/28.1.27, 10592173 PMC102409

[ref12] Kasem E. Kurihara T. Tabuchi K. (2018). Neurexins and neuropsychiatric disorders. Neurosci. Res. 127, 53–60. doi: 10.1016/j.neures.2017.10.012, 29221905

[ref13] Kelai S. Maussion G. Noble F. Boni C. Ramoz N. Moalic J. M. . (2008). Nrxn3 upregulation in the globus pallidus of mice developing cocaine addiction. Neuroreport 19, 751–755. doi: 10.1097/WNR.0b013e3282fda231, 18418251 PMC4710739

[ref14] Kim K. Picciotto M. R. (2023). Nicotine addiction: more than just dopamine. Curr. Opin. Neurobiol. 83:102797. doi: 10.1016/j.conb.2023.102797, 37832393 PMC10842238

[ref9015] Korsunsky I. Millard N. Fan J. Slowikowski K. Zhang F. Wei K. . (2019). Fast, sensitive and accurate integration of single-cell data with harmony. Nat. Methods 16, 1289–1296. doi: 10.1038/s41592-019-0619-0, 31740819 PMC6884693

[ref16] Liu X. Hua F. Yang D. Lin Y. Zhang L. Ying J. . (2022). Roles of neuroligins in central nervous system development: focus on glial neuroligins and neuron neuroligins. J. Transl. Med. 20:418. doi: 10.1186/s12967-022-03625-y, 36088343 PMC9463862

[ref17] Liu C. Tose A. J. Verharen J. P. H. Zhu Y. Tang L. W. de Jong J. W. . (2022). An inhibitory brainstem input to dopamine neurons encodes nicotine aversion. Neuron 110, 3018–3035.e7. doi: 10.1016/j.neuron.2022.07.003, 35921846 PMC9509462

[ref18] Morales M. Margolis E. B. (2017). Ventral tegmental area: cellular heterogeneity, connectivity and behaviour. Nat. Rev. Neurosci. 18, 73–85. doi: 10.1038/nrn.2016.165 28053327

[ref19] Nguyen C. Mondoloni S. le Borgne T. Centeno I. Come M. Jehl J. . (2021). Nicotine inhibits the VTA-to-amygdala dopamine pathway to promote anxiety. Neuron 109, 2604–2615.e9. doi: 10.1016/j.neuron.2021.06.013, 34242565

[ref20] Novak G. Boukhadra J. Shaikh S. A. Kennedy J. L. Le Foll B. (2009). Association of a polymorphism in the NRXN3 gene with the degree of smoking in schizophrenia: a preliminary study. World J. Biol. Psychiatry 10, 929–935. doi: 10.1080/15622970903079499 19658047

[ref21] Nussbaum J. Xu Q. Payne T. J. Ma J. Z. Huang W. Gelernter J. . (2008). Significant association of the neurexin-1 gene (NRXN1) with nicotine dependence in European and African-American smokers. Hum. Mol. Genet. 17, 1569–1577. doi: 10.1093/hmg/ddn044, 18270208 PMC2902291

[ref22] Oriol L. Chao M. Kollman G. J. Dowlat D. S. Singhal S. M. Steinkellner T. . (2025). Ventral tegmental area interneurons revisited: GABA and glutamate projection neurons make local synapses. eLife 13:RP100085. doi: 10.7554/eLife.100085, 40238649 PMC12002793

[ref23] Phillips R. A. Tuscher J. J. Black S. L. Andraka E. Fitzgerald N. D. Ianov L. . (2022). An atlas of transcriptionally defined cell populations in the rat ventral tegmental area. Cell Rep. 39:110616. doi: 10.1016/j.celrep.2022.110616 PMC10888206 35385745

[ref24] Picciotto M. R. Kenny P. J. (2021). Mechanisms of nicotine addiction. Cold Spring Harb. Perspect. Med. 11:a039610. doi: 10.1101/cshperspect.a039610, 32341069 PMC8091956

[ref25] Poulin J. F. Gaertner Z. Moreno-Ramos O. A. Awatramani R. (2020). Classification of midbrain dopamine neurons using single-cell gene expression profiling approaches. Trends Neurosci. 43, 155–169. doi: 10.1016/j.tins.2020.01.004, 32101709 PMC7285906

[ref26] Prévost E. D. Ward L. A. Alas D. Aimale G. Ikenberry S. Fox K. . (2025). Untangling dopamine and glutamate in the ventral tegmental area. bioRxiv 25:640201. doi: 10.1101/2025.02.25.640201

[ref27] Qi J. Zhang S. Wang H. L. Barker D. J. Miranda-Barrientos J. Morales M. (2016). VTA glutamatergic inputs to nucleus accumbens drive aversion by acting on GABAergic interneurons. Nat. Neurosci. 19, 725–733. doi: 10.1038/nn.4281, 27019014 PMC4846550

[ref28] Song J. Y. Ichtchenko K. Südhof T. C. Brose N. (1999). Neuroligin 1 is a postsynaptic cell-adhesion molecule of excitatory synapses. Proc. Natl. Acad. Sci. USA 96, 1100–1105. doi: 10.1073/pnas.96.3.1100, 9927700 PMC15357

[ref29] Südhof T. C. (2008). Neuroligins and neurexins link synaptic function to cognitive disease. Nature 455, 903–911. doi: 10.1038/nature07456, 18923512 PMC2673233

[ref30] Südhof T. C. (2017). Synaptic Neurexin complexes: a molecular code for the logic of neural circuits. Cell 171, 745–769. doi: 10.1016/j.cell.2017.10.024, 29100073 PMC5694349

[ref31] Tiklová K. Björklund Å. K. Lahti L. Fiorenzano A. Nolbrant S. Gillberg L. . (2019). Single-cell RNA sequencing reveals midbrain dopamine neuron diversity emerging during mouse brain development. Nat. Commun. 10:581. doi: 10.1038/s41467-019-08453-1, 30718509 PMC6362095

[ref32] Wang Y. Yang Z. Shi X. Han H. Li A. N. Zhang B. . (2025). Investigating the effect of Arvcf reveals an essential role on regulating the mesolimbic dopamine signaling-mediated nicotine reward. Commun. Biol. 8:429. doi: 10.1038/s42003-025-07837-y, 40082601 PMC11906728

[ref33] Wills L. Ables J. L. Braunscheidel K. M. Caligiuri S. P. B. Elayouby K. S. Fillinger C. . (2022). Neurobiological mechanisms of nicotine reward and aversion. Pharmacol. Rev. 74, 271–310. doi: 10.1124/pharmrev.121.000299, 35017179 PMC11060337

[ref34] Wittenberg R. E. Wolfman S. L. De Biasi M. Dani J. A. (2020). Nicotinic acetylcholine receptors and nicotine addiction: a brief introduction. Neuropharmacology 177:108256. doi: 10.1016/j.neuropharm.2020.108256, 32738308 PMC7554201

[ref35] Yoo J. H. Zell V. Gutierrez-Reed N. Wu J. Ressler R. Shenasa M. A. . (2016). Ventral tegmental area glutamate neurons co-release GABA and promote positive reinforcement. Nat. Commun. 7:13697. doi: 10.1038/ncomms13697, 27976722 PMC5171775

[ref36] Zell V. Steinkellner T. Hollon N. G. Warlow S. M. Souter E. Faget L. . (2020). VTA glutamate neuron activity drives positive reinforcement absent dopamine co-release. Neuron 107, 864–873.e4. doi: 10.1016/j.neuron.2020.06.011, 32610039 PMC7780844

